# Successful endovascular embolization of the common hepatic artery for pseudoaneurysm associated with pancreatic fistula after liver transplantation: a case report

**DOI:** 10.1186/s40792-023-01723-7

**Published:** 2023-08-10

**Authors:** Kazuki Sasaki, Tadafumi Asaoka, Shogo Kobayashi, Yoshifumi Iwagami, Daisaku Yamada, Yoshito Tomimaru, Takehiro Noda, Hiroshi Wada, Kunihito Gotoh, Hidenori Takahashi, Noboru Maeda, Yasushi Kimura, Yusuke Ono, Yuichiro Doki, Hidetoshi Eguchi

**Affiliations:** 1 Department of Gastroenterological Surgery, Graduate School of Medicine, Osaka University, 2-2 E2 Yamadaoka, Suita, Osaka 565-0871 Japan; 2 Department of Gastroenterological Surgery, Osaka Police Hospital, Osaka, Japan; 3 Department of Gastroenterological Surgery, Osaka International Cancer Institute, Osaka, Japan; 4 Department of Surgery, National Hospital Organization Osaka National Hospital, Osaka, Japan; 5 Department of Diagnostic and Interventional Radiology, Osaka International Cancer Institute, Osaka, Japan; 6 Department of Diagnostic and Interventional Radiology, Osaka University Graduate School of Medicine, Suita, Japan

**Keywords:** Hepatic artery pseudoaneurysm, Orthotopic liver transplantation, Endovascular treatment, *n*-2-Butyl-cyanoacrylate

## Abstract

**Background:**

After orthotopic liver transplantation (OLT), complications such as hepatic artery stenosis, thrombosis, and bleeding are possible. Hepatic artery pseudoaneurysms (HAP) are prone to rupture, rupture hemorrhage, and increased mortality risk. Endovascular treatment of HAP may result in recurrence, even after successful embolization with thrombin. Formation of a HAP in the common hepatic artery (CHA) is challenging because the CHA is the only artery in the liver graft after OLT. Therefore, CHA embolization in HAP is not an initial option. We report a case of HAP at the CHA after OLT that was treated with endovascular therapy, resulting in the occlusion of the CHA with coil embolization, achieving a radical cure.

**Case presentation:**

A 59-year-old man with decompensated hepatitis C virus cirrhosis underwent deceased donor whole-liver transplantation after graft failure of a living donor liver transplantation. After the second transplantation, the patient developed infectious narrow-necked HAP at the CHA associated with postoperative pancreatic fistula. Repeated transcatheter arterial embolization with thrombin and *n*-butyl-2-cyanoacrylate was unsuccessful, as confirmed by postprocedure angiography, which revealed recanalization and regrowth of the HAP. Eight months after the first transcatheter arterial embolization, the patient presented with a chief complaint of abdominal pain due to an enlarged HAP. Angiography of the superior mesenteric artery (SMA) revealed a collateral bypass around the bile duct from the SMA to the liver graft. Coil embolization of the HAP in the CHA completely occluded the HAP without complications. More than 2 years after coil embolization, the liver graft function test results remained within normal limits without HAP recurrence.

**Conclusions:**

HAP at the CHA after liver transplantation can be fatal if ruptured. Because the liver is a highly angiogenic organ, even if initial treatment is not successful, radical treatment to occlude the CHA with HAP is possible if sufficient collateral vessels are developed.

**Supplementary Information:**

The online version contains supplementary material available at 10.1186/s40792-023-01723-7.

## Introduction

Orthotopic liver transplantation (OLT) is practiced worldwide and is regarded as a standard procedure for end-stage liver disease, with 10,418 transplants performed through December 2020 in Japan [1]. Despite improvements in surgical techniques and immunological maintenance, severe biliary and vascular complications can occur after OLT, leading to graft failure and patient death.

Hepatic artery-related complications such as hepatic artery thrombosis (HAT), stenosis (HAS), and pseudoaneurysm (HAP) are rare but can occur after OLT. Their incidences are 3.5%, 2–13%, and 1.1–2.5% for HAT, HAS, and HAP, respectively [2–4]. Among these, HAP is the most serious complication [2, 5], with the risk of sudden life-threatening rupture that leads to graft loss and high mortality (53–69%) [4]. HAP after OLT often presents with nonspecific symptoms such as fever, abdominal discomfort, and gastrointestinal bleeding. Early diagnosis of HAP through close monitoring is crucial to prevent life-threatening hemorrhages with shock and high mortality. The location of the HAP depends on its etiology. Intrahepatic HAP is related to iatrogenic injuries, such as percutaneous transhepatic intervention or liver biopsy. Extrahepatic HAP is associated with anastomotic problems, local infection, bilio-enteric anastomosis [4], bile leakage, or postoperative pancreatic fistula.

Therapeutic approaches for HAP include surgical management and interventional radiology. Surgical treatments include HAP resection, ligation, and subsequent retransplantation or bypass with a saphenous vein graft [6], autologous radial artery, or inferior mesenteric artery [7]. For endovascular treatment, coil embolism, covered stent, endovascular balloon occlusion [8], and embolic agents, such as thrombin and *n*-butyl-2-cyanoacrylate (NBCA) are used [9, 10]. Surgery has traditionally been the treatment for HAP after OLT; however, minimally invasive interventional radiological approaches have recently become common [3, 11]. However, in some cases, the patient’s condition and the HAP location make simultaneous graft stenting and arterial embolization difficult.

A particular challenge is the treatment of a HAP formed at the common hepatic artery (CHA) because the CHA is usually the only arterial blood supply pathway to the liver graft. Therefore, CHA embolization in cases of HAP is not an initial option. We report a case of HAP in the CHA after liver transplantation. Although recurrence of HAP was observed after initial treatment with NBCA and thrombin infusion, coil embolization of the CHA with HAP was finally performed. A radical cure was achieved due to the formation of collateral flow to the liver graft via the superior mesenteric artery (SMA) that developed during that period.

## Case presentation

A 59-year-old male with decompensated hepatitis C virus cirrhosis (Child–Pugh score of 10 [C], and a Model for End-stage Liver Disease score of 31) underwent living donor liver transplantation (LDLT) using a left liver plus caudate lobe graft from his spouse. The patient developed partial liver infarction because of a portal vein thrombus extending from the main to the left portal branches, leading to liver graft failure. After that, the patient underwent deceased donor liver transplantation (DDLT) using a whole-liver graft on postoperative day 43 (Additional file 1: Fig. S1). A portal vein was reconstructed between the recipient’s superior mesenteric vein and the graft portal vein with the donor’s left common iliac vein graft interposition using end-to-end anastomosis. The biliary duct was constructed using duct-to-duct anastomosis.

**Fig. 1:**
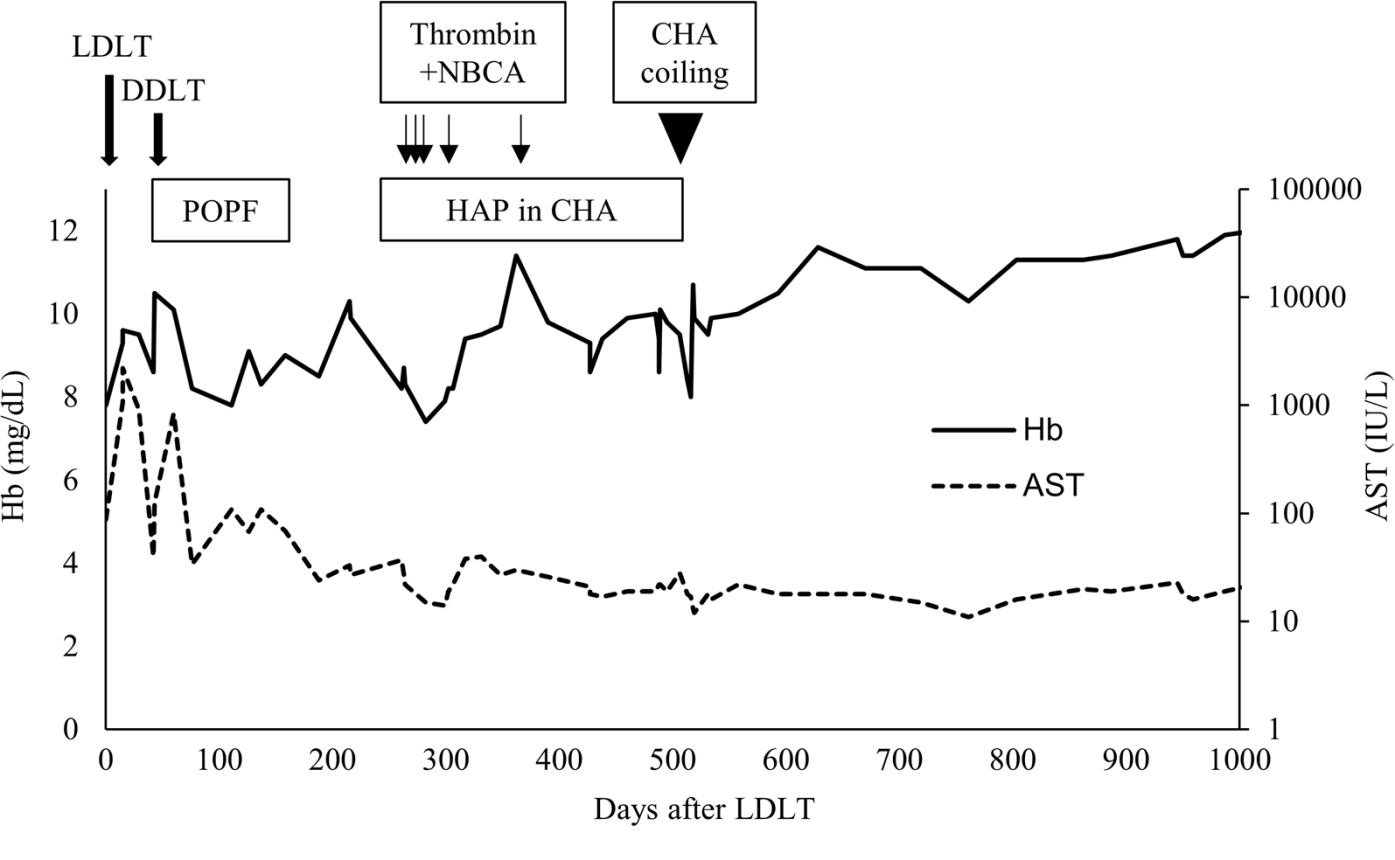
The postoperative course of the patient. One month after LDLT, the patient underwent DDLT due to liver graft failure resulting from a portal vein thrombus extending from the main to the left portal branch. Approximately 8 months after LDLT, a HAP developed in the CHA and was controlled by thrombin and NBCA injections; however, the HAP was recanalized repeatedly. Finally, coil embolization was performed on the HAP and CHA, and the patient survived recurrence-free for more than 500 days. *AST* aspartate aminotransferase, *CHA* common hepatic artery, *DDLT* deceased donor liver transplantation, *HAP* hepatic artery pseudoaneurysm, *LDLT* living donor liver transplantation, *NBCA* *n*-2-butyl-cyanoacrylate, *POPF* postoperative pancreatic fistula

After DDLT, aspartate aminotransferase levels decreased immediately (Fig. 1), but the patient developed a postoperative pancreatic fistula (grade B, according to the International Study Group of Pancreatic Fistula). *Candida* species were cultured in the fluid drainage, and the fistula was relieved by conservative treatment. Seven months after the surgery, the patient was transferred to a nursing hospital.

**Fig. 2:**
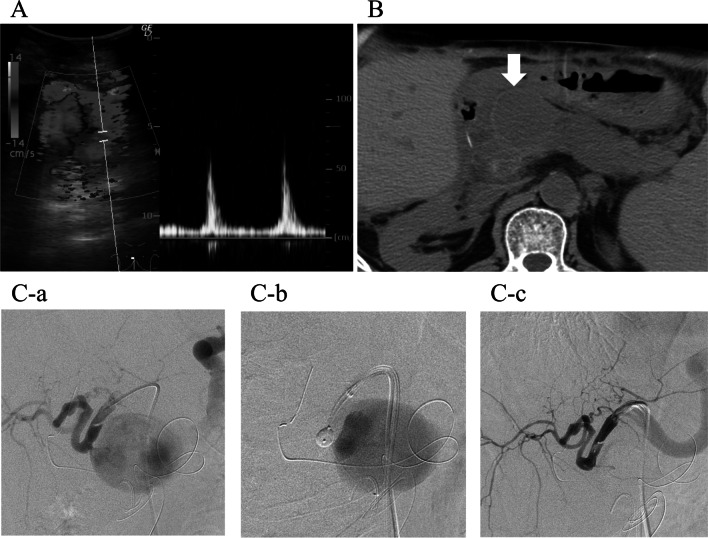
Diagnosis of the HAP in the CHA 8 months after LDLT. **A** Abdominal ultrasonography revealing a 4-cm mass with a to-and-fro wave pattern at the neck of the lesion on Doppler. **B** Plain abdominal computed tomography imaging showing a 4-cm cystic lesion in the area of the pancreatic head. **C**-**a** Celiac angiography revealing a narrow-neck type HAP in the CHA and a tortuous PHA. **C**-**b** Under balloon protection of the PHA, thrombin was injected into the HAP. **C**-**c** Post-procedure angiography showing complete occlusion of the HAP and a good flow to the graft liver. *CHA* common hepatic artery, *HAP* hepatic artery pseudoaneurysm, *LDLT* living donor liver transplantation, *PHA* proper hepatic artery

One month after the transfer (postoperative day 261), the patient experienced acute abdominal discomfort around the epigastrium. Abdominal computed tomography revealed a 4-cm cystic lesion in the pancreatic head (Fig. 2A). Abdominal ultrasonography revealed a to-and-fro wave pattern in the cyst (Fig. 2B). Angiography of the celiac artery confirmed HAP in the CHA (Fig. 2C-a). Because the HAP had a narrow neck shape, thrombin (800 U) was injected under balloon protection of the proper hepatic artery (PHA) (Fig. 2C-b). After this procedure, the HAP disappeared, and the hepatic artery was patent (Fig. 2C-c). Daily doppler abdominal ultrasonography was performed. On the third day, doppler abdominal ultrasonography showed that the blood flow resumed in the mass. Embolization was performed with thrombin under IVR (Interventional Radiology). However, during the third relapse, the HAP was recanalized repeatedly and occluded by embolization with NBCA (1:2 NBCA: Lipiodol). No collateral formation was observed at this point.

**Fig. 3:**
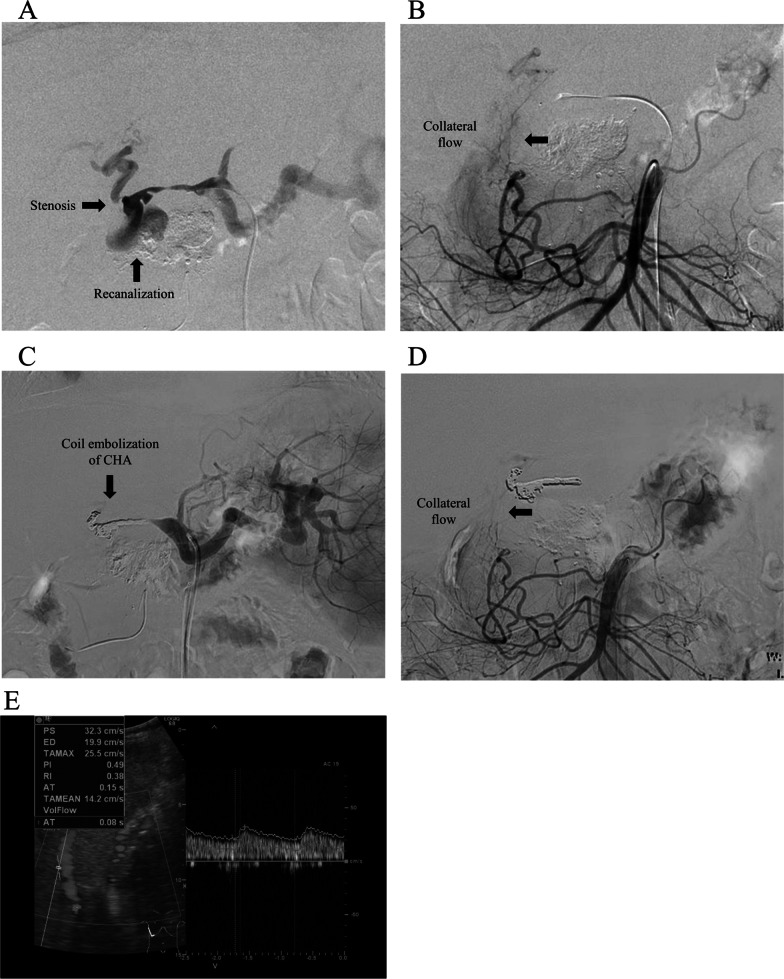
Coil embolization of the HAP in the CHA. Coil embolization of the recanalized HAP 8 months after the first endovascular treatment. **A** Celiac angiogram of the HAP in the CHA. The HAP neck had widened, and the diameter of the PHA had narrowed compared to the first endovascular treatment. **B** SMA angiography image of the collateral vascular network around the bile duct (arrow). **C** Coil embolization performed on the HAP and CHA. **D** Post-procedural angiography of the SMA showing preserved flow to the graft via a vascular network around the bile duct. **E** Abdominal ultrasonography showing the intrahepatic arterial waveforms after coil embolization of the CHA. *CHA* common hepatic artery, *HAP* hepatic artery pseudoaneurysm, *PHA* proper hepatic artery, *SMA* superior mesenteric artery

The HAP resolved for a while. However, abdominal pain and fever were later observed, thus confirming the fifth HAP recurrence 8 months after the first thrombin infusion (postoperative day 517). Angiography showed that the neck of the HAP had widened to 1 cm, and the PHA diameter had narrowed (Fig. 3A). Another NBCA injection into the HAP or stent graft was unsuitable because of the widened neck and the small diameter of the PHA. Fortunately, the liver graft was enhanced by a collateral bypass around the bile duct from the SMA (Fig. 3B) and under from left gastric vein with balloon occlusion of CHA (Additional file 2: Fig. S2). Given the collateral vasculature; we believe CHA embolization is safe. Coil embolization (Target and Penumbra) of the HAP and CHA under balloon protection was performed (Fig. 3C). After embolization, angiography confirmed complete HAP occlusion and blood flow to the liver graft through the SMA (Fig. 3D). Additionally, abdominal ultrasonography showed that the intrahepatic arterial waveforms were visible after coil embolization (Fig. 3E). After that, the patient’s clinical course was uneventful. Liver function test results remained within normal limits. The patient was discharged on day 20 after coil embolization and has remained stable without abscess or HAP recurrence for over 2 years.

## Discussion

Along with HAT, HAS, portal vein thrombosis, and portal vein stenosis, HAP is another vascular complication that can occur after OLT [12]. Although HAPs are rare, they can lead to severe outcomes, including graft loss and even death. Therefore, early detection and prompt treatment of HAP before rupture are crucial.

Table 1 summarizes reports of the treatment of extrahepatic pseudoaneurysms after liver transplantation over the past 10 years (from 2013 to 2022). Because there have been several types of cases, including ruptured and unruptured HAP, it is difficult to uniformly compare the treatments to determine which is best. Surgical treatment includes revascularization, hepatic artery (HA) ligation, and repeat liver transplantation. Boleslawski showed the benefit of HA ligation for ruptured HAP with high mortality [13]. Among 17 patients with ruptured HAP, 6 of 10 of those patients with HA ligation achieved long-term survival. Interestingly, some patients who achieved long-term survival without repeat transplantation underwent HA ligation without ischemic bile duct injury. Regarding endovascular therapy, the usefulness of endovascular therapies such as coil embolization and stent grafting has been reported [3, 4, 9, 14]. Kadohisa et al. reported three cases of ruptured HAP treated with embolization [14]. The HA was completely occluded. One patient was successfully treated with autologous vein graft, and another was successfully treated with liver transplantation; however, one patient died of liver abscess and sepsis while waiting for the formation of collateral channels.

**Table 1:** Case series regarding extrahepatic HAP after liver transplantation 2013–2022

Year	Author	N	Onset (day) median, range	Clinical presentation (n)	Surgical treatment (n)	Endovascular therapy (n)	None (n)	Outcome alive/total
2013	Saad	20	48, 1–1098	Hypotension (12) GI-bleeding (7) Sepsis (2) Dropped hematocrit without symptoms (1)	Surgery (2)			1/2
						Embolization (4) (selective embolization (1))		1/4
						Stent-graft (8)(+ ReOLT (1))		6/8
					ReOLT (5)			5/5
							None (1)	0/1
2013	Panaro	9	39.6, 22–92	Bleeding (4), fever (3) asymptomatic (3)	Revascularization with saphenous bypass (9)(+ ReOLT (1))			8/9
2013	Boleslawski	17	29, 2–92	Hemo peritoneum (10) GI-bleeding (5) Hemobilia (1) Hematoma (1)	Anastomosis revision (5)(+ ReOLT (1))			1/5
					HA ligation (10)(+ ReOLT (3))			6/10
						Embolization (1)		0/1
							None (1)	0/1
2014	Volpin	16	13, 4–100	Shock (10) GI-bleeding (4) Bleed through drains (5) Abdominal pain (8)	Excision + revascularization (7)			4/7
					Ligation (5)(+ ReOLT (1))			2/5
						Embolization (1)		1/1
						Covered stent (1)		1/1
							None (2)	0/2
2017	Thorat	2^a^	7, 2.5 Mo	Bleeding (2)		Stent graft (2)		2/2
2022	Kadohisa	3	41, 68, 19 Mo	GI-bleeding (3)		Embolization (3) (+ revascularization (1) + ReOLT(1))		2/3
2023	This case	1	7 Mo	Abdominal pain, fever		Embolization (1)		1/1

^a^Extrahepatic HAP cases were extracted from original reports. *Mo* Month, *ReOLT* re-orthotopic liver transplantation, *HA* hepatic artery

In our case, HAP in the CHA after liver transplantation was successfully treated with CHA embolization because of the collateral vasculature to the liver graft that developed around the bile duct. HAP generally develops after OLT, where a local infection has injured the artery wall at the anastomotic site because of technical difficulties during anastomosis completion or due to iatrogenic effects from procedures such as angioplasty for arterial stenosis. In this case, a HAP developed in the CHA and not at the anastomotic site. The retrospective imaging evaluation revealed a portal vein thrombus during DDLT, which required a jump graft through the SMV to the back of the pancreas in the presence of severe adhesions. The GDA was considered to have been sacrificed during dissection around the CHA for hemostasis. In fact, the GDA was visible on angiography before DDLT, but it was not visible on postoperative day 14 (Additional file 1: Fig. S1). And this, combined with the postoperative pancreatic fistula, may have led to the development of HAP at the GDA stump.

NBCA is generally a permanent embolic reagent that polymerizes in the blood. It is injected in a mixture with Lipiodol for the embolization of pseudoaneurysms [15], aneurysms [16], arteriovenous malformations [17], and varices. The advantage of NBCA over coil embolization is that it can be applied to tortuous vessels, unlike coil embolization. However, recanalization can occur after injection [18]. In our case, we initially did not perform coil embolization because of the risk of occluding the native PHA, leading to graft ischemia. We also did not choose a stent graft because a suitable size was unavailable owing to the tortuous artery. Thus, we chose thrombin and NBCA as initial treatments. Despite the disappearance of HAP immediately after injection, NBCA could have been washed out by the high arterial pressure of the CHA during the ensuing months. Because NBCA was not a definitive treatment in the present case, we finally switched to coil embolization of the HAP in the CHA.

Neovascularization of the liver is often observed after OLT, especially when HAT is involved [19–22]. There have been few reports of HAP treatment that involves waiting for the formation of collateral channels. According to Panaro et al., the mean interval between the diagnosis of hepatic artery thrombosis and neovascularization of the liver was 4.1 months (range, 3–5.5 months) [20]. Four factors have been cited in the development of neovascularization after DDLT: late hepatic artery thrombosis (> 30 days), early hepatic artery stenosis (< 30 days), thrombosis at the anastomotic site, and Roux-en-Y anastomosis [20]. The trigger for neovascularization is unknown; however, the assumed mechanism is that prolonged liver hypoxia due to HAT or stenosis induces the expression of vascular endothelial growth factor and hypoxia-inducible factor 1-α, leading to angiogenesis from the surrounding tissue to the liver [22]. Neovascularization has been reported in various vessels, including the omentum and mesenteric, subcostal, lumbar, renal, and iliac arteries [20, 22]. The connective tissue around the common bile duct and the hilar plate is usually preserved during donor hepatectomy to ensure sufficient blood flow to the bile duct after transplantation. In this patient, 8 months after the first thrombin embolization (517 days after LDLT), we confirmed collateral formation around the bile duct; however, we did not know the timing of the collateral vessel development. We do know that during the third relapse (286 days after the LDLT), it was not confirmed from SMA angiography (data not shown). We speculate that arterial stenosis and repeated thrombin or NBCA injections may have led to chronic ischemia of the liver, inducing neovascularization.

## Conclusions

This case illustrates the need to pay special attention to patients with risk factors for HAP after OLT, such as local infections. In such cases, regular ultrasound or computed tomography imaging should be performed to enable the early recognition of HAP and management of this life-threatening condition. Neovascularizing the liver after OLT could benefit graft survival by allowing the embolizing of a HAP in the CHA if needed. Fortunately, in this case, because of the collateral bypass formed around the bile duct more than 500 days after LDLT, HAP in the CHA was cured without complications by endovascular treatment with NBCA, thrombin, and coil embolization.

### Supplementary Information

**Additional file 1: Fig. S1.** The hypothesized location of the HAP in the CHA. A) Drawing before DDLT (a), after DDLT (b) and 7 months after DDLT(c). Before DDLT, portal vein thrombosis was observed. During DDLT, PV reconstructed using the donor’s left common iliac vein graft interposition from the SMV, passed from the back of the pancreas to the head and anastomosed with the donor portal vein. During this process, GDA was sacrificed. Postoperative pancreatic fistula developed. Seven months after DDLT, a pseudoaneurysm formed at the GDA stump. B Angiogram before DDLT showing GDA, CHA, and PHA (a). Maximum Intensity Projection (MIP) image of POD14 (b) and POD49 (c). 3D constructed contrast-enhanced CT image 7 months after DDLT (d). The GDA seen preoperatively is obscured after DDLT. A hepatic pseudoaneurysm was detected on the GDA stump. CHA: common hepatic artery, PHA: proper hepatic artery, GDA: gastroduodenal artery.

**Additional file 2: Fig. S2.** Angiography prior to coil embolization of the HAP in the CHA. A. Angiography from LGA with balloon occlusion of CHA showed intrahepatic artery via collateral tract. BAngiography from SMA showed intrahepatic artery via IPDA and peribiliary collateral tract. Arrows indicate intrahepatic arteries via collateral channels. LGA: left gastric artery, IPDA: inferior pancreaticoduodenal artery.

## Data Availability

Data sharing is not applicable to this article as no datasets were generated or analyzed during the current study.

## Abbreviations

HAP: Hepatic artery pseudoaneurysm
CHA: Common hepatic artery
OLT: Orthotopic liver transplantation
SMA: Superior mesenteric artery
NBCA: *N*-Butyl-2-cyanoacrylate
LDLT: Living donor liver transplantation
DDLT: Deceased donor liver transplantation
PHA: Proper hepatic artery
AST: Aspartate aminotransferase
POPF: Postoperative pancreatic fistula
IVR: Interventional radiology
