# Supplementary figures and images for: Successful endovascular embolization of the common hepatic artery for pseudoaneurysm associated with pancreatic fistula after liver transplantation: a case report
Source: Surg Case Rep. 2023 Aug 10;9:143. doi: 10.1186/s40792-023-01723-7 (PMC10415245; doi:10.1186/s40792-023-01723-7)

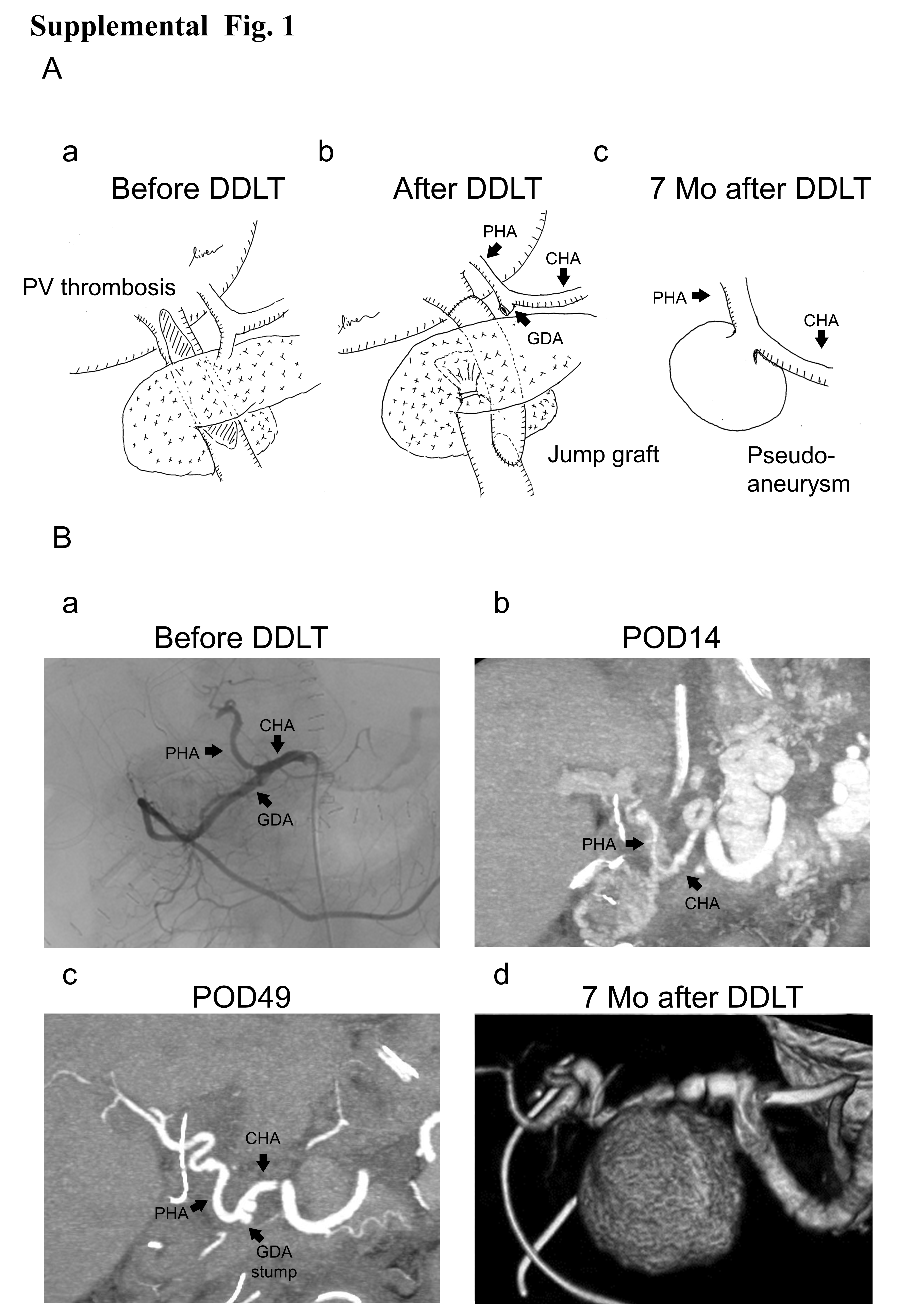

Supplement: Supplementary file 1 — Additional file 1: Fig. S1. The hypothesized location of the HAP in the CHA. A) Drawing before DDLT (a), after DDLT (b) and 7 months after DDLT(c). Before DDLT, portal vein thrombosis was observed. During DDLT, PV reconstructed using the donor’s left common iliac vein graft interposition from the SMV, passed from the back of the pancreas to the head and anastomosed with the donor portal vein. During this process, GDA was sacrificed. Postoperative pancreatic fistula developed. Seven months after DDLT, a pseudoaneurysm formed at the GDA stump. B Angiogram before DDLT showing GDA, CHA, and PHA (a). Maximum Intensity Projection (MIP) image of POD14 (b) and POD49 (c). 3D constructed contrast-enhanced CT image 7 months after DDLT (d). The GDA seen preoperatively is obscured after DDLT. A hepatic pseudoaneurysm was detected on the GDA stump. CHA: common hepatic artery, PHA: proper hepatic artery, GDA: gastroduodenal artery. [file 40792_2023_1723_MOESM1_ESM.tif]

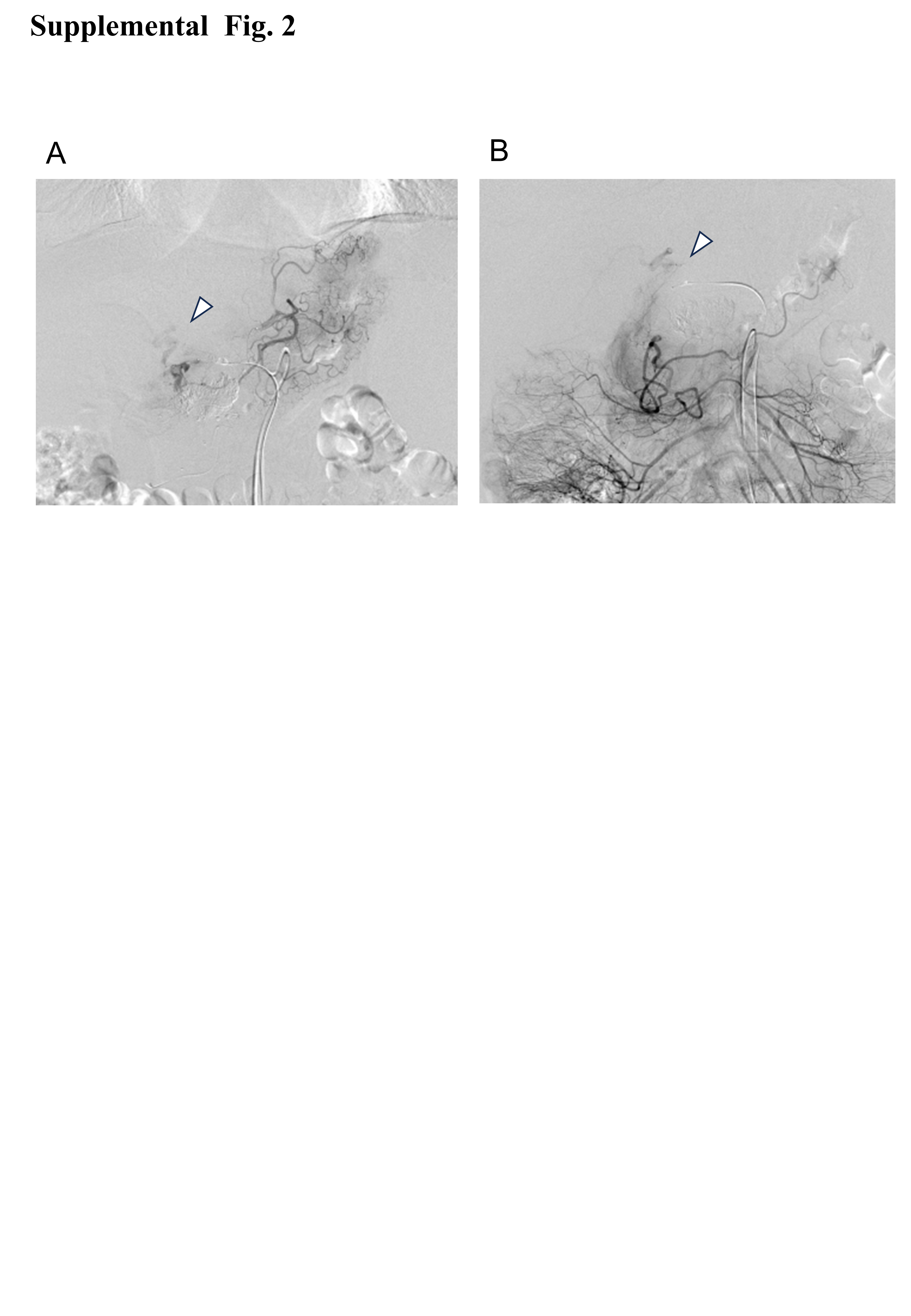

Supplement: Supplementary file 2 — Additional file 2: Fig. S2. Angiography prior to coil embolization of the HAP in the CHA. A. Angiography from LGA with balloon occlusion of CHA showed intrahepatic artery via collateral tract. BAngiography from SMA showed intrahepatic artery via IPDA and peribiliary collateral tract. Arrows indicate intrahepatic arteries via collateral channels. LGA: left gastric artery, IPDA: inferior pancreaticoduodenal artery. [file 40792_2023_1723_MOESM2_ESM.tif]
